# Metabolomic profile of glycolysis and the pentose phosphate pathway identifies the central role of glucose-6-phosphate dehydrogenase in clear cell-renal cell carcinoma

**DOI:** 10.18632/oncotarget.3823

**Published:** 2015-04-14

**Authors:** Giuseppe Lucarelli, Vanessa Galleggiante, Monica Rutigliano, Francesca Sanguedolce, Simona Cagiano, Pantaleo Bufo, Gaetano Lastilla, Eugenio Maiorano, Domenico Ribatti, Andrea Giglio, Grazia Serino, Antonio Vavallo, Carlo Bettocchi, Francesco Paolo Selvaggi, Michele Battaglia, Pasquale Ditonno

**Affiliations:** ^1^ Department of Emergency and Organ Transplantation-Urology, Andrology and Kidney Transplantation Unit, University of Bari, Bari, Italy; ^2^ Department of Pathology, University of Foggia, Foggia, Italy; ^3^ Department of Pathology, University of Bari, Bari, Italy; ^4^ Department of Basic Medical Sciences, Neurosciences and Sensory Organs, University of Bari, Bari, Italy; ^5^ National Cancer Institute “Giovanni Paolo II”, Bari, Italy

**Keywords:** renal cell carcinoma, metabolomics, glycolysis, pentose phosphate pathway, G6PDH

## Abstract

The analysis of cancer metabolome has shown that proliferating tumor cells require a large quantities of different nutrients in order to support their high rate of proliferation. In this study we analyzed the metabolic profile of glycolysis and the pentose phosphate pathway (PPP) in human clear cell-renal cell carcinoma (ccRCC) and evaluate the role of these pathways in sustaining cell proliferation, maintenance of NADPH levels, and production of reactive oxygen species (ROS). Metabolomic analysis showed a clear signature of increased glucose uptake and utilization in ccRCC tumor samples. Elevated levels of glucose-6-phosphate dehydrogenase (G6PDH) in association with higher levels of PPP-derived metabolites, suggested a prominent role of this pathway in RCC-associated metabolic alterations. G6PDH inhibition, caused a significant decrease in cancer cell survival, a decrease in NADPH levels, and an increased production of ROS, suggesting that the PPP plays an important role in the regulation of ccRCC redox homeostasis. Patients with high levels of glycolytic enzymes had reduced progression-free and cancer-specific survivals as compared to subjects with low levels. Our data suggest that oncogenic signaling pathways may promote ccRCC through rerouting the sugar metabolism. Blocking the flux through this pathway may serve as a novel therapeutic target.

## INTRODUCTION

Renal cell carcinoma (RCC) accounts for approximately 3% of all adult malignancies. Recent estimates have calculated that in 2014, 63,920 new cases will be diagnosed and 13,860 patients will die of RCC in the United States [1]. Although nowadays RCC can be diagnosed at an early stage, up to 30% of the patients present metastatic disease at diagnosis, and around 20-30% of subjects undergoing surgery will suffer recurrence [2, 3].

In recent years there has been a growing interest in identifying tumor markers not only for diagnostic purposes but also to improve the predictive power of clinical and pathological parameters [4]. A prognostic role has been proposed for several circulating biomarkers associated with different features of RCC biology, including carbonic anhydrase IX (CAIX), hypoxia-inducible factor-1α (HIF1α), CA15-3, and C-reactive protein (CRP) [5-8]. The progressive introduction of high-throughput technologies has led to a greater understanding of molecular mechanisms underlying the development of cancer and the identification of novel potential therapeutic targets. In this scenario, analysis of the cancer metabolome has shown that proliferating tumor cells require large quantities of different nutrients to support their high rate of proliferation [9, 10]. For this purpose, the cancer cell metabolism undergoes adaptive changes that include a switch from oxidative phosphorylation to glycolysis and increased lactate production, for example. However, recent studies have shown that in addition to the so-called Warburg effect (i.e. the shift to aerobic glycolysis with lactate production), alternative glucose metabolic pathways can be activated in order to generate the building blocks required for cancer cell growth [11-13]. Among these, the pentose phosphate pathway (PPP) is an alternative metabolic pathway that, starting from glucose-6-phosphate, generates precursors for nucleotide biosynthesis and NADPH for anabolic reactions and redox homeostasis maintenance [14, 15]. Renal cell carcinoma is fundamentally a metabolic disease [16]. Many studies suggest that an altered metabolism is involved in the development of RCC [17, 18]. Moreover, many genes implicated in RCC pathogenesis play an important role in controlling cell metabolism [19, 20]. Here we analyze the metabolic profile of glycolysis and the pentose phosphate pathway in human clear cell-renal cell carcinoma (ccRCC), and what role these play in sustaining cell proliferation, the maintenance of NADPH levels, production of reactive oxygen species (ROS) and in reducing cisplatin-induced cytotoxicity. In addition, we assess the role of some key metabolic enzymes as prognostic factors for RCC progression and survival.

## RESULTS

### Glucose metabolism and the pentose phosphate pathway (PPP) are perturbed in clear cell RCC

**Figure 1:**
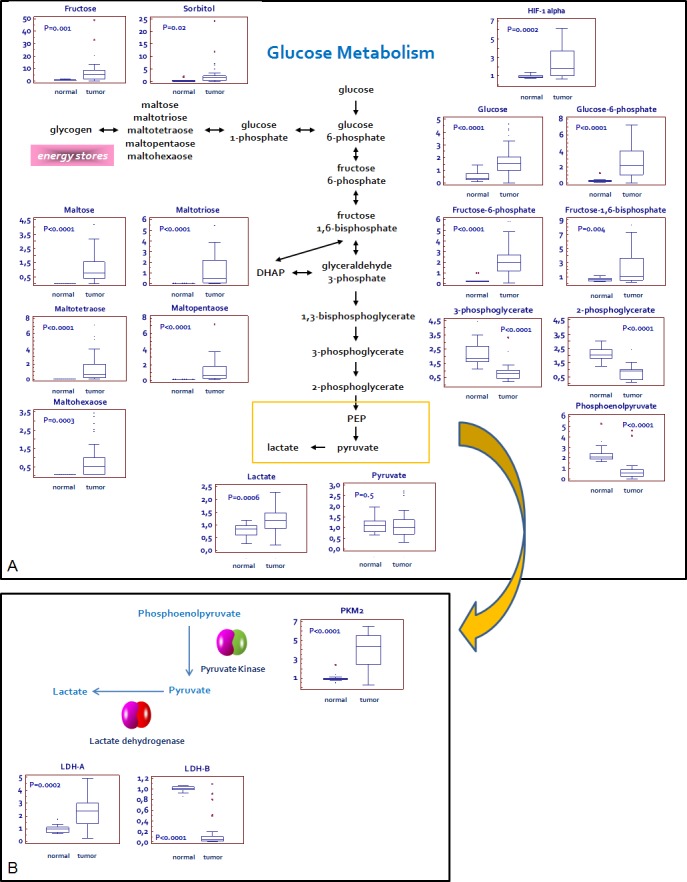
Schematic model summarizing the differences in glucose metabolism between normal and renal tumor cells Significant elevations in glucose and related sugars, along with higher levels of upstream glycolytic intermediates, lower levels of downstream glycolytic intermediates, and high levels of lactate are indicative of an increased glucose utilization through glycolysis in pathological samples **A.**The Pyruvate kinase isoform M2 (PKM2) and L-lactate dehydrogenase A chain (LDH-A), but not L-lactate dehydrogenase A chain (LDH-B), are over-expressed in tumor as compared to normal tissue. This expression profile is consistent with the increased lactate levels observed in RCC **B.** Y-axis: metabolite relative amount.

A clear signature of increased glucose uptake and utilization was observed in ccRCC tumor samples, as glucose was significantly elevated, along with higher levels of other sugars (fructose and sorbitol) and their phosphate derivatives (Figure 1A). Glycogenolysis was also accelerated in pathological samples, significant elevations in the glycogen synthesis/degradation products being observed. In particular, maltose, maltotriose, maltotetraose, maltopentaose, and maltohexaose were significantly elevated in tumor tissue. The increased glucose availability was accompanied by an increase of upstream glycolytic intermediates (glucose 6-phosphate, fructose 6-phosphate and fructose-1,6-bisphosphate), reduction in downstream intermediates (3-phosphoglycerate, 2-phosphoglycerate, and phosphoenolpyruvate) and increased lactate production.

**Figure 2:**
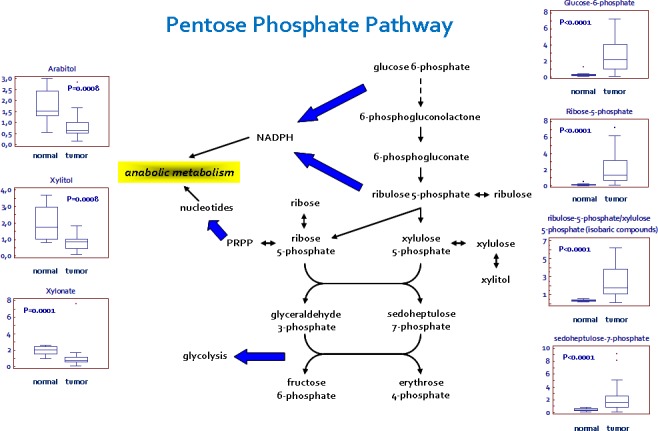
Schematic model summarizing the differences in the pentose phosphate pathway (PPP) between normal and renal tumor cells Significant elevations in PPP intermediates and depletions in pentitols are suggestive of an increased glucose utilization through this pathway to support nucleotide biosynthesis and NADPH production. Y-axis: metabolite relative amount.

In addition to the observed changes in glycolytic intermediates and lactate, metabolites reflective of PPP activity were also altered in the pathological tissues (Figure 2). Apparent elevations in glucose utilization through the PPP were observed in tumor tissue, as evidenced by higher levels of the intermediates sedoheptulose 7-phosphate, ribose 5-phosphate, and ribulose 5-phosphate/xylulose 5-phosphate (isobaric compounds). A significant reduction in several pentitols produced from these intermediates, such as arabitol, xylitol, and xylonate, was observed in the pathological samples, suggestive of an increased PPP activity for the generation of nucleotides.

### Glycolytic enzymes expression and glucose-6-phosphate dehydrogenase activity are increased in tumor tissue

**Figure 3:**
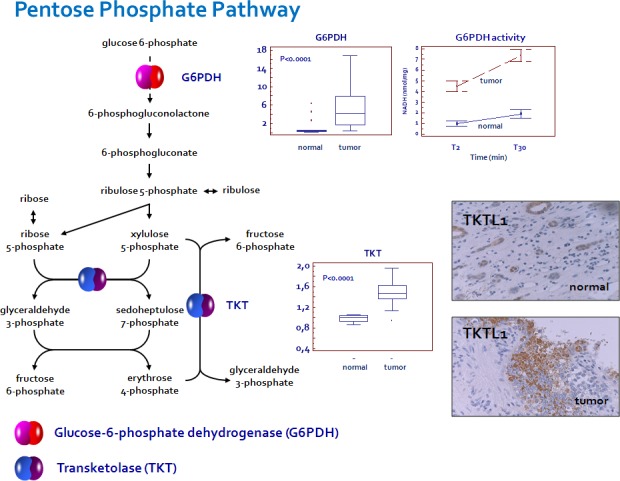
Glucose-6-phosphate dehydrogenase (G6PDH) and transketolase (TKT) are over-expressed in ccRCC An increased enzymatic activity of G6PDH was observed in renal cancer as compared with normal tissue. Transketolase-like-1 (TKTL1) immunoreactivity was restricted to tumor cells, while the surrounding stromal tissue showed no staining. In normal tissue the protein was detected only in the cytoplasm of some tubular epithelial cells.

Analysis of the glycolytic enzymes Glucose-6-phosphate isomerase/Autocrine motility factor (G6PI/AMF), L-lactate dehydrogenase A chain (LDH-A), Pyruvate kinase isoform M2 (PKM2) and Transketolase (TKT) demonstrated an increased expression of all these proteins in RCC as compared with non neoplastic tissue (Figure 1B and 3). Conversely, the levels of L-lactate dehydrogenase B chain (LDH-B) were significantly higher in normal kidney tissue and the inverse LDH-A/LDH-B ratio between normal and neoplastic tissue was in accordance with the higher efficiency lactate production observed in ccRCC (Figure 1B).

Taken together, these findings are consistent with the observation of significantly higher Hypoxia-inducible factor 1-α (HIF-1α) levels in tumor tissues (*P* = 0.0002; Figure 1), indicating that HIF-1α drives the up-regulation of these metabolic enzymes.

In addition, since in some tumors it has been observed that the specific effects of TKT activity were due to over-expression of the transketolase-like-1 (TKTL1) protein, we performed IHC on our tissue samples [21,22]. We found the expression of this protein in 24 (40%) tumor specimens. The immunoreactivity was restricted to tumor cells, while the surrounding stromal tissue showed no staining. Immunohistochemical analysis of normal tissue showed TKTL1 expression only in 1 (5%) case, where the protein was detected in the cytoplasm of some tubular epithelial cells (Figure 3).

Since Glucose-6-phosphate dehydrogenase (G6PDH) is the rate-limiting enzyme of the pentose phosphate pathway, we studied its expression and enzymatic activity. We observed elevated levels of G6PDH and an increased activity of this enzyme in renal cancer as compared with normal tissue (Figure 3). These findings, in association with the increased expression of TKT and higher levels of PPP-derived metabolites, suggest a prominent role of this pathway in RCC-associated metabolic alterations.

### Glucose-6-phosphate isomerase (Autocrine motility factor) and its receptor (AMFR) are over-expressed in clear cell RCC

**Figure 4:**
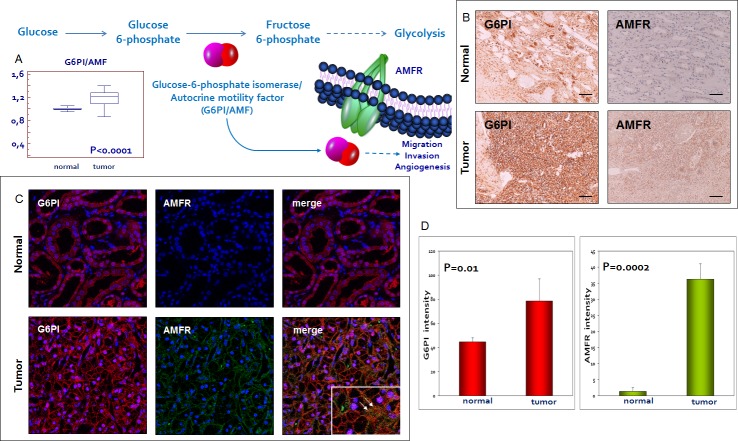
Glucose-6-phosphate isomerase/autocrine motility factor (G6PI/AMF) and its receptor (AMFR) are over-expressed in RCC G6PI tissue levels are increased in pathological samples as compared with normal tissue **A.** Immunohistochemistry, immunoﬂuorescence and confocal laser scanning microscopy demonstrated an increased expression of G6PI and AMFR in tumor tissue. G6PI and AMFR co-localization was noted on the cell membrane **B**-**C.** Fluorescence intensity quantization for G6PI and AMFR **D.** Arrows indicate G6PI/AMFR co-localization. Original magnifications (20x, 63X), scale bar = 100 μm.

**Figure 5:**
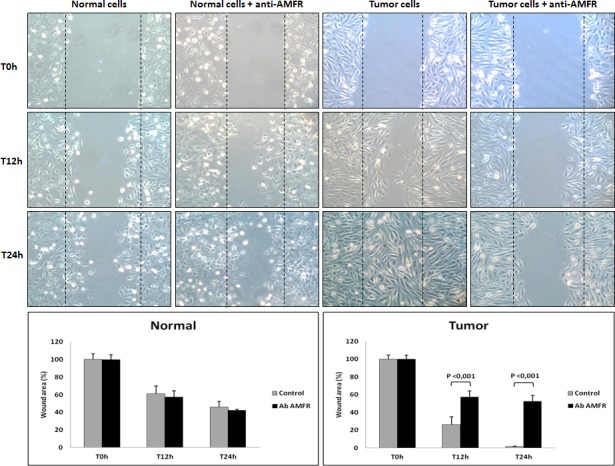
Wounded normal and tumor cell monolayers were photographed 12 and 24 hours after the mechanical scratch and the area of the wounds was measured in 3-independent wound sites per group When specified, the cells were exposed to 2ng/μl of antibody anti-AMFR for 30 minutes. RCC cells treated with anti-AMFR antibody had decreased cell migratory capabilities compared to untreated tumor cells.

**Figure 6:**
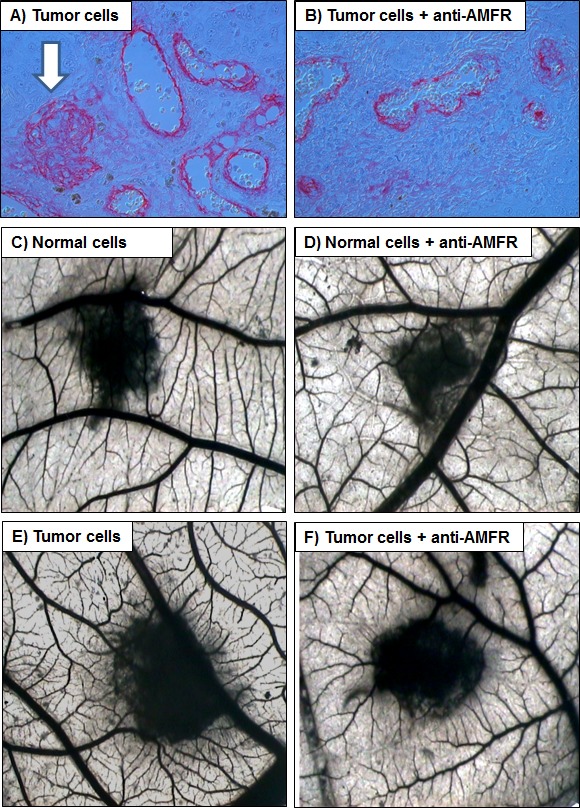
Chick embryo chorioallantoic membrane (CAM) invasion and angiogenic assays **A, B.** The number of tumor cells treated with anti-AMFR antibody invading the CAM mesenchyme was significantly lower when compared with untreated tumor cells (arrow). **C**-**F.** Macroscopic pictures of CAMs at day 12 of incubation, showing gelatin sponges containing normal **C**-**D.** and tumor cells **E**-**F.** When tumor cell were treated with 2ng/μl of antibody anti-AMFR, a lower vascular reaction was detectable around the silicon ring **F.** while untreated tumor cells were surrounded by allantoic vessels developing radially toward it in a ‘spoked-wheel’ pattern (in E). Original magnifications (50X).

We firstly performed immunohistochemistry on normal and pathological tissues, to visualize the location and expression of G6PI and AMFR. Normal kidney showed weak staining for G6PI, predominantly localized in the renal tubule cell cytoplasm. Instead, pathological tissue showed a stronger staining in cancer cells, with a prevalently membranous pattern. Similarly, AMFR expression was very weak in normal kidney, but showed higher levels in ccRCC. To confirm these findings we evaluated G6PI/AMFR co-expression in the normal and neoplastic renal tissue samples. In particular, immunofluorescence staining showed an increased signal for both G6PI and AMFR in cancer cells, and their co-localization on plasma membrane (Figure 4). To evaluate the role of G6PI/AMFR axis in renal cancer cell migration, invasion and angiogenesis, *in vitro* and *in vivo* assays were performed. Scratch wound healing assay and chick embryo chorioallantoic membrane (CAM) invasion assay showed that RCC cells treated with anti-AMFR antibody had decreased cell migratory and invasive capabilities (Figure 5 and 6). In particular, to investigate the invasive capacity of tumor cells alone or treated with anti-AMFR antibody, cell suspensions were seeded on the top of the chick embryo CAM and their ability to cross the CAM epithelium and to invade the underlying mesenchyme was evaluated by histological means. As shown in Figure 6, the number of tumor cells treated with anti-AMFR antibody invading the CAM mesenchyme was significantly lower when compared with untreated tumor cells (mean ± SD = 50±5 *vs* 7±2; *P* = 0.001).

Moreover CAM assay showed that that gelatin sponges soaked with normal and tumor cells suspension were surrounded by numerous allantoic vessels that developed radially toward the implant in a spoked wheel pattern (mean ± SD= 12 ± 3 and 24 ± 3 blood vessels, respectively; *P* = 0.001). In contrast, few blood vessels were identified around sponges containing tumor cells treated with anti-AMFR antibody (mean ± SD= 12 ± 3; *P* = 0.001 *vs* untreated tumor cells).

### G6PDH is required for renal cancer cell survival and maintenance of NADPH levels

**Figure 7:**
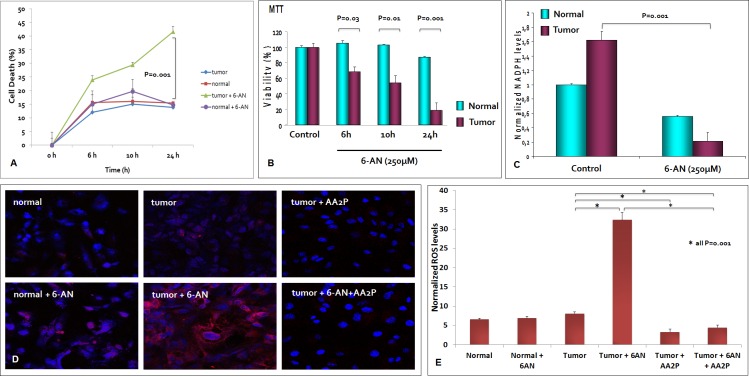
G6PDH is required for renal cancer cell survival and redox homeostasis maintenance At 6, 10 and 24 hours, the death rate of cancer cells pretreated with 6-aminonicotinamide (6-AN) was signiﬁcantly higher than that of unblocked tumor cells **A.** The MTT assay revealed a decreased cell viability when tumor cells were pretreated with 6-AN 250μM **B.** G6PDH inhibition by 6-AN pre-treatment caused a significant decrease in NADPH levels **C.** and an increased reactive oxygen species (ROS) production in renal cancer cells **D, E.** The exposure to ascorbic acid 2-phosphate (AA2P) significantly reduced the production of ROS in cancer cells pre-treated with 6-AN **D, E.**

We hypothesized that G6PDH could have an important role in renal cancer cell proliferation and in NADPH production. To test this hypothesis we blocked G6PDH activity in primary normal and tumor cell lines using 6-aminonicotinamide (6-AN), a competitive inhibitor of G6PDH. We used a previously defined inhibitor concentration that was confirmed in preliminary dose-response experiments (data not shown). We observed, in pre-treated cancer cells, a significant reduction in cell survival as compared to normal cells (*P* = 0.001) (Figure 7A). The MTT assay conﬁrmed these ﬁndings, demonstrating a decreased cell viability when cancer cells were pretreated with 6-AN (53.9% at 10 and 19.2% at 24 hours, Figure 7B). To rule out the potential off-target effects of 6-AN, we complemented the experiments using small interfering RNA (siRNA) targeting G6PDH. We confirmed silencing of G6PDH by real time PCR in normal and tumor renal cells, and showed that molecular inhibition of G6PDH decreased cancer cell survival (Supplementary Figure 1 and 2).

Moreover, G6PDH inhibition caused a significant decrease in NADPH levels, suggesting that ccRCC requires an active G6PDH for efficient NADPH production (Figure 7C). Next, we investigated whether G6PDH inhibition could differently alter ROS levels in normal and neoplastic cells. As shown in other tumors, 6-AN treatment increased ROS production especially in cancer cells, suggesting that the PPP, through the production of reducing agents such as NADPH, plays an important role in the regulation of RCC redox homeostasis (Figure 7D and 7E). The exposure to ascorbic acid 2-phosphate (AA2P), a stable vitamin C derivative, dramatically reduced the production of ROS in cancer cells pre-treated with 6-AN (Figure 7D and 7E).

### G6PDH decreases cisplatin induced renal cancer cell death

**Figure 8:**
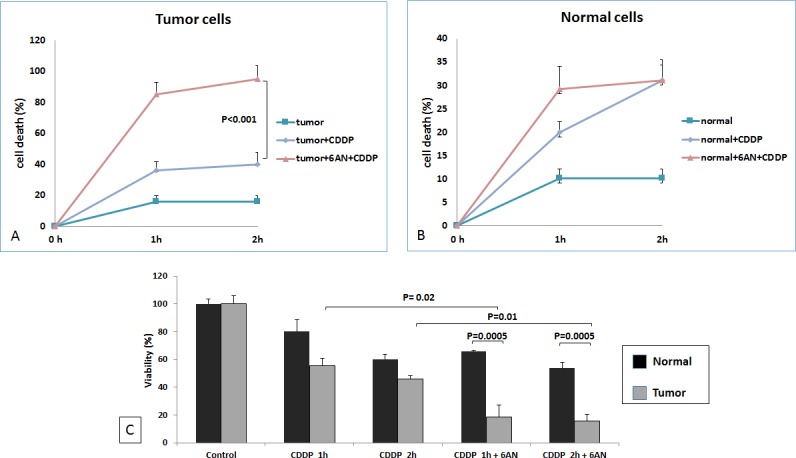
G6PDH has role in RCC resistance to cisplatin-induced cytotoxicity At 1 and 2 hours (h) death rate of blocked tumor cells (tumor+6AN+CDDP) was significantly higher than that of unblocked cells (tumor+CDDP) (*P* < 0.0001) **A.** No difference was observed between blocked (tumor+6AN+CDDP) and unblocked (tumor+CDDP) normal cells **B.** MTT assay revealed significantly decreased cell viability when renal tumor cells were pretreated with 6-AN before cisplatin incubation **C.**

We evaluated the role of G6PDH in reducing cisplatin-induced cytotoxicity in ccRCC. Normal and renal cancer cells were pretreated with 6-AN. After cisplatin treatment the death rate of tumor cells treated with 6-AN was significantly greater than that of untreated cancer cells (*p* < 0.0001, Figure 8). MTT assay confirmed these findings by demonstrating decreased cell viability when tumor cells were pretreated with 6-AN before cisplatin incubation (18.3% at 1h and 15.8% at 2 h) (Figure 8).

### High levels of G6PI, TKT and G6PDH are associated with reduced progression-free and cancer specific survival in patients with clear cell RCC

**Figure 9:**
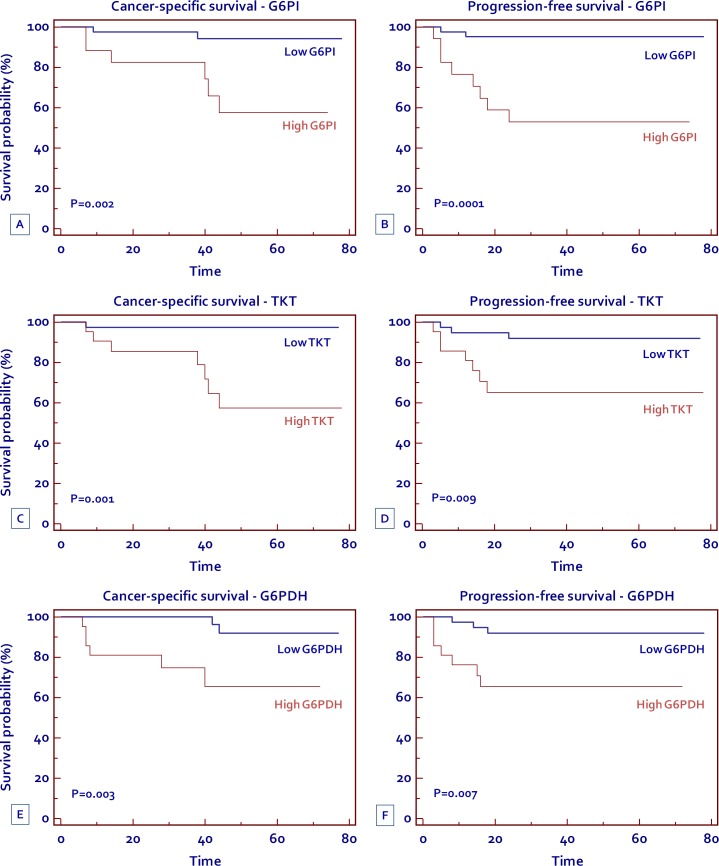
Kaplan-Meier cancer-specific survival (CSS) and progression-free survival (PFS) curves, stratified by G6PI (A, B) TKT (C, D) and G6PDH (E, F) tissue levels

To evaluate the association between patients survival and the expression levels of G6PI, TKT and G6PDH, we classified the entire population by high versus low expression levels according to the cut-offs provided by Receiver Operating Characteristic (ROC) curve analysis. A fold change of 1.5, 1.5 and 5, was selected for G6PI, TKT and G6PDH, respectively. Kaplan-Meier survival curves for cancer-specific survival (CSS) and progression-free survival (PFS), stratified by the three proteins, are shown in Figure 9. Both CSS and PFS were significantly decreased in patients with high levels of G6PI, TKT and G6PDH.

Spearman's test showed a positive correlation between G6PI (r_s_= 0.53; *P* = 0.001), TKT (r_s_= 0.47; *P* = 0.003) and G6PDH (r_s_= 0.58; *P* = 0.001) levels and the tumor stage. No correlation was found between protein levels and the Fuhrman grade.

## DISCUSSION

Metabolic reprogramming is one of the hallmarks of cancer [23]. In particular, several studies have shown how the alterations in cancer cell metabolism are not focused on maximizing ATP production, but rather on providing the building blocks needed to sustain macromolecular synthesis [10, 12, 24]. In RCC, HIF1α reprograms the metabolism of cancer cells, in part by increasing the glucose uptake, and in part by controlling the glucose flux through the glycolytic and pentose phosphate pathways [25, 26]. In fact, in tumor tissue we observed high levels of glucose and other sugars, in association with an increase in upstream glycolytic intermediates (glucose 6-phosphate and fructose 6-phosphate), and reduction in downstream intermediates (3-phosphoglycerate, 2-phosphoglycerate, and phosphoenolpyruvate).

We did not find statistically significant differences in pyruvate levels between pathological and normal tissue and this observation might seem paradoxical considering the higher expression of PKM2 in these highly glycolytic cells. It is well known that although PKM2 has a lower enzymatic activity and an increased sensitivity to inhibition than the PKM1 isoform, PKM2-expressing cancer cells produce more glucose-derived pyruvate than normal cells expressing PKM1 [27, 28]. It has been suggested that PKM2 could provide an advantage to cancer cell proliferation by slowing glycolysis and facilitating anabolic metabolism. In fact, the reduced conversion of phosphoenolpyruvate (PEP) to pyruvate leads to an accumulation of upstream glycolytic intermediates and their subsequent shunting into anabolic pathways such as PPP, glycerol and serine/glycine synthesis.

In addition, other studies have proposed the existence of alternative pathways for pyruvate production involving the activity of other enzymes such as serine dehydratase and phosphoglycerate mutase [29].

The increased lactate production observed in ccRCC, as well as in many other tumors, is another paradox of the glucose metabolism in cancer. In fact, it is surprising that the decreased activity of PKM2 is associated with increased lactate levels, considering that lactate is produced directly from pyruvate. These observations can be explained by the particular LDH isoenzyme expressed in cancer cells [30].

LDH is a tetramer composed of two different subunits, LDH-A and LDH-B, which assemble to produce five isoforms of this enzyme: LDH-1 (LDH-B4), LDH-2 (LDH-A1B3), LDH-3 (LDH-A2B2), LDH-4 (LDH-A3B1), and LDH-5 (LDH-A4). In our study we found a reduced expression of LDH-B and higher levels of LDH-A in cancer tissue. Thus, low levels of LDH-B can be expected to reduce the LDH isoenzymes 1 to 4, and increase the levels of LDH-5, which is the isoenzyme with the highest efficiency lactate production, particularly under hypoxic conditions.

Another interesting finding was the higher expression of G6PI and its receptor in tumor tissue. Glucose-6-phosphate isomerase (G6PI), also known as phosphoglucose isomerase, was initially identified as the second glycolytic enzyme that catalyzes the interconversion of glucose-6-phosphate to fructose-6-phosphate. Later studies demonstrated that G6PI was the same as the autocrine motility factor (AMF) and neuroleukin. We now know that GPI/AMF is involved in tumor cell migration, invasion and angiogenesis, and that it mediates its biological effects through the interaction with its surface receptor (AMFR/gp78) [31, 32]. Since G6PI protein levels were found to be increased in tumor tissue, we proceeded with immunohistochemical and immunofluorescence analyses. Both evaluations confirmed an increased expression of G6PI in tumor tissues compared to normal specimens. Moreover, in accordance with its biological role, G6PI was also identified outside the neoplastic cells and co-staining with AMFR showed the co-localization with its membrane receptor. AMFR was seen in both normal and neoplastic cells, even if normal epithelial tubular cells stained very weakly for this protein. In addition, *in vitro* and *in vivo* assays showed that AMFR was implicated in renal cancer cell migration, invasion, and tumor angiogenesis.

Taken together, these findings in association with high levels of PPP enzymes (G6PDH and TKT) and metabolic intermediates (sedoheptulose 7-phosphate, ribose 5-phosphate, and ribulose 5-phosphate/xylulose 5-phosphate), suggest that a rerouting of the sugar metabolism toward the PPP occurs, with the aim of promoting both anabolic reactions and redox homeostasis in RCC.

In particular, ribose-5-phosphate is a sugar used in the synthesis of nucleotides, therefore the increased flux of metabolites through the PPP should provide an advantage in cell growth and survival. Since G6PDH is the rate-limiting enzyme of the PPP, we studied the effects of its inhibition by 6-AN [33]. G6PDH was expressed at higher levels in renal cancer cells, in which it showed an increased activity compared to normal cells. Accordingly, the inhibition of G6PDH caused a significant decrease in cancer cell growth, confirming the importance of this pathway in RCC. Another important byproduct of PPP is NADPH, a reducing compound that is used for biosynthetic reactions and redox state control. 6-AN pre-treatment induced a significant reduction in NADPH levels and an increased production of ROS in renal cancer cells, suggesting that G6PDH, and consequently the PPP, have a fundamental role in maintaining redox homeostasis in RCC. Cytotoxic chemotherapy has been largely ineffective in RCC. In recent years, a better understanding of the molecular basis of RCC has led to introduction of anti-angiogenic therapies for this tumor, although these drugs yield partial responses in a minority of patients, with no evidence of complete responses [34]. We hypothesized that PPP could be implicated in RCC chemoresistance. To explore this hypothesis renal cancer cells were pretreated with 6-AN. After cisplatin incubation the cell death rate of RCC cells treated with 6-AN was significantly higher than that of unblocked cancer cells.

Finally, we analyzed the prognostic role of G6PI, TKT and G6PDH, depending on their tissue expression levels. Kaplan-Meier curves showed significant differences in CSS and PFS among groups of patients with high versus low protein expression. In particular, patients with high levels of G6PI, TKT and G6PDH had a 5-year survival rate of 57.6%, 57.4%, and 65.4%, respectively, as compared to 94.2%, 97.3%, and 92% for subjects with low levels. Similar findings were observed for PFS (52.9%, 65.1%, and 65.3% versus 95.1%, 91.9%, and 91.9% for G6PI, TKT and G6PDH, respectively). In conclusion our data suggest that in clear cell-RCC, oncogenic signaling pathways may promote cancer through rerouting the sugar metabolism. In particular, the flux of sugars through the PPP, in association with the upregulation of G6PDH, promote both anabolic reactions and redox homeostasis. Blocking the flux through this pathway may serve as a novel therapeutic target.

## MATERIALS AND METHODS

### Study population and tissue collection

Primary renal tumor (*n* = 60) and non neoplastic tissues (*n* = 20) were collected from 60 patients who underwent radical or partial nephrectomy for ccRCC. All specimens were immediately used to obtain primary cell cultures after frozen-section confirmation of the diagnosis. Two pathologists confirmed the presence of clear cell RCC in the neoplastic tissues and excluded tumor cells in the healthy specimens. Detailed clinical and pathological characteristics of the patients are summarized in Table 1. All patients were preoperatively staged by thoraco-abdominal Computed Tomography (CT) or Magnetic Resonance Imaging. Tumor staging was reassigned according to the 7th edition of the AJCC-UICC TNM classification. The 2004 World Health Organization and Fuhrman classifications were used to attribute histological type and nuclear grade, respectively. Written informed consent to take part was given by all participants. The protocol for the research project has been approved by local Ethics Committee and it conforms to the provisions of the Declaration of Helsinki in 1995.

**Table 1:** Clinical and pathological characteristics

variable	n=60
Age (years) median range	51 26-84
Gender Male Female	36 (60%) 24 (40%)
Dimensions (cm) median range	5 2 −12.5
Pathological stage pT1a pT1b pT2 pT3	18 (30%) 22 (36.6%) 8 (13.4%) 12 (20%)
pN+	4 (6.6%)
cM+	8 (13.3%)
Fuhrman grade G1-2 G3-4	40 (66.7%) 20 (33.3%)
Follow-up Median (months) 95% CI	42.5 38-60

### Metabolite analysis

#### Sample preparation

All tissue samples were maintained at −80°C until processed. The sample preparation process was carried out using the automated MicroLab STAR® system (Hamilton Company). Recovery standards were added prior to the first step in the extraction process for quality control purposes. Sample preparation was conducted using a proprietary series of organic and aqueous extractions to remove the protein fraction while allowing maximum recovery of small molecules. The resulting extract was divided into two fractions; one for analysis by Liquid chromatography/Mass Spectrometry (LC/MS) and one for analysis by Gas chromatography/Mass Spectrometry (GC/MS). Samples were placed briefly on a TurboVap® (Zymark) to remove the organic solvent. Each sample was frozen and vacuum dried. Samples were then prepared for the appropriate investigation, namely LC/MS or GC/MS.

#### Liquid chromatography/mass spectrometry (LC/MS, LC/MS2)

The LC/MS portion of the platform was based on a Waters ACQUITY UPLC and a Thermo-Finnigan LTQ mass spectrometer, which consisted of an electrospray ionization (ESI) source and linear ion-trap (LIT) mass analyzer. The sample extract was split into two aliquots, dried, then reconstituted in acidic or basic LC-compatible solvents, each of which contained 11 or more injection standards at fixed concentrations. One aliquot was analyzed using acidic positive-ion optimized conditions and the other using basic negative-ion optimized conditions, in two independent injections using separate dedicated columns. Extracts reconstituted in acidic conditions were gradient-eluted using water and methanol, both containing 0.1% Formic acid, while the basic extracts, which also used water/methanol, contained 6.5mM Ammonium Bicarbonate. MS analyses alternated between MS and data-dependent MS^2^ scans using dynamic exclusion.

#### Gas chromatography/mass spectrometry (GC/MS)

The samples destined for GC/MS analysis were re-dried under vacuum desiccation for a minimum of 24 hours prior to being derivatized under dried nitrogen using bistrimethyl-silyl-trifluoroacetamide (BSTFA). The GC column was 5% phenyl and the temperature ramp ranged from 40° to 300° C over a 16 minute period. Samples were analyzed on a Thermo-Finnigan Trace DSQ fast-scanning single-quadrupole mass spectrometer using electron impact ionization. The instrument was tuned and calibrated for mass resolution and mass accuracy on a daily basis. The information output from the raw data files was automatically extracted as discussed below.

#### Accurate mass determination and MS/MS fragmentation (LC/MS), (LC/MS/MS)

The LC/MS portion of the platform was based on a Waters ACQUITY UPLC and a Thermo-Finnigan LTQ-FT mass spectrometer, which had a linear ion-trap (LIT) front end and a Fourier transform ion cyclotron resonance (FT-ICR) mass spectrometer backend. For ion counts exceeding 2 million, an accurate mass measurement could be performed. Accurate mass measurements could be made on the parent ion as well as fragments. The typical mass error was less than 5 ppm. The characterization of ion counts of less than two million requires a greater amount of effort. Fragmentation spectra (MS/MS) were typically generated in a data-dependent manner but if necessary, targeted MS/MS could be employed, as in the case of lower level signals.

#### Compound identification

Compounds were identified by comparison with library entries of purified standards or recurrent unknown entities. The identification of known chemical entities was based on comparison with metabolomic library entries of purified standards.

#### Normalization

a data normalization step was performed to correct variation resulting from instrument inter-day tuning differences. Essentially, each compound was corrected in run-day blocks by registering the medians to equal one (1.00) and normalizing each data point proportionately.

### Preparation of tissue extracts

100 mg of healthy and pathologic tissue samples were rapidly homogenized with 2.0 mL ice-cold Cell Lysis Buffer containing freshly added phosphatase and protease inhibitors (Millipore). Tissue homogenates were incubated on ice for 30 minutes with occasional vortexing and centrifuged at 14,000 × g for 20 minutes at 4–8°C. The tissue extracts were immediately aliquoted and the protein concentration was determined with the Coomassie Bradford assay.

### Primary culture of human kidney epithelial and tumor cells

Tumor (ccRCC) and normal kidney tissue specimens were immediately placed in a Petri dish with phosphate-buffered saline (PBS) 1x and cut into small pieces of about 1 mm^3^. Each small piece of tissue was placed on the surface of the Petri dish, previously wetted with 1ml of Dulbecco's Modified Eagle Medium (DMEM, Invitrogen) supplemented with 10% fetal bovine serum (FBS, Sigma-Aldrich) and 1% penicillin-streptomycin-L-glutamine (Sigma-Aldrich). The Petri dish was maintained for 2 hours at 37°C in a humidified atmosphere with 5% CO_2_. After this time, the small specimens were covered with 4 ml of DMEM and placed back in the incubator. Cell proliferation was obtained around the kidney specimens. Kidney epithelial tubular and neoplastic cells were isolated with EpCAM (CD326) Ab-conjugated magnetic microbeads (Miltenyi Biotec, Bergisch Gladbach, Germany) under the effect of a magnetic field generated by the Mini MACS Separation Unit (Miltenyi Biotec), as previously described [35].

### MILLIPLEX^®^ MAP human glycolysis pathway magnetic bead panel assays

The MILLIPLEX^®^ MAP Human Glycolysis Pathway Magnetic Bead Panel (HGPMAG-27K, Millipore) was applied in 96-well plates for the simultaneous quantification of the following proteins in tissue lysates: G6PI/AMF (Glucose-6-phosphate isomerase/Autocrine motility factor), LDHA (L-lactate dehydrogenase A chain), LDHB (L-lactate dehydrogenase B chain), PKM2 (Pyruvate kinase isoform M2) and TKT (Transketolase), and HIF-1α (Hypoxia-inducible factor 1-α).

For the immunoassay procedures, 25μl of each dilute lysate sample in Assay Buffer (5μg total protein/well) and HeLa cells lysate (positive control) were added into wells in duplicate, according to the manufacturer's instructions. To each well, 25 μl of the Mixed Beads were added and the plate was incubated for 2 hours at room temperature. Human glycolysis pathway detection biotinylated antibodies were added for 1 hour; each captured a specific bead. After that, the reaction mixture was incubated for 30 minutes with Streptavidin-PE conjugate to complete the reaction on the surface of each microsphere. Finally, the MILLIPLEX^®^ MAP was analyzed by Luminex xMAP^®^ technology. The immunoassay on the surface of each fluorescent-coded magnetic bead, MagPlex-C microsphere, was identified and quantified based on fluorescent signals. The median fluorescence intensity (MFI) was read with the Luminex 200^TM^ instrument and measured with xPONENT^®^ software.

### Determination of glucose-6-phosphate dehydrogenase activity

Glucose-6-phosphate dehydrogenase (G6PDH) activity was assessed in tissue lysates using the Glucose-6-Phosphate Dehydrogenase Assay Kit (Abcam, Cambridge, UK). The assay was performed by adding 50μl of dilute samples (1:2 with assay buffer) into duplicate wells of 96-well plates. Then, 50 μl of Reaction Mix (G6PDH substrate and G6PDH developer) were added to each well containing samples or positive control. At two (T1) and thirty (T2) minutes after addition of the reagents and incubation at 37°C, the plate was read in an ELISA microplate reader at 450 nm. G6PDH activity was spectrophotometrically measured by monitoring the production of NADH, and calculated as a change of optical density (ΔA_450nm_) at T1 and T2 per sample volume added into the reaction well, expressed in nmol/mg.

### Immunohistochemistry (IHC)

Immunohistochemical evaluation of G6PI, autocrine motility factor receptor (AMFR), and transketolase-like-1 (TKTL1) protein expression was carried out on paraffin-embedded tissue sections of kidney specimens. Thin (5 μm) sections were deparaffinized and rehydrated using xylene and ethanol. After antigen retrieval, by microwave in citrate buffer 0.01M pH=6.0, the sections were incubated with 3% H2O2 for 10 min to block endogenous peroxidase activity. The sections were blocked with serum-free protein block (Dako, Glostrup, Denmark) at room temperature (RT) for 10 min and then incubated with anti-G6PI (1:200, Novus Biologicals, Littleton, CO, USA) and anti-TKTL1 antibodies (1:500, Novus Biologicals) at 4°C overnight, and with anti-AMFR (1:100, Novus Biologicals) for 2 h at room temperature. Binding of the secondary biotinylated antibody was detected by the Dako Real EnVision Detection System, Peroxidase/DAB kit (Dako), according to the manufacturer's instructions. Sections were counterstained with Mayer haematoxylin and mounted with glycerol (Dako Cytomation). Serial sections incubated with blocking solution omitting primary antibodıes were used as negatıve controls. Images were taken at 40X magnification with a Leica microscope equipped with a Coolpix 990 digital camera (Nikon, Calenzano, Italy).

### Immunofluorescence and confocal laser scanning microscopy

Paraffin-embedded kidney sections were double-stained for G6PI (1B7D7, Novus Biologicals, Littleton, CO, USA) and AMFR (Novus Biologicals). The expression and localization of proteins was evaluated by indirect immunofluorescence and confocal microscopy analysis. After antigen unmasking, the sections were blocked with 2% BSA in PBS for 1 h at room temperature. Sections were incubated overnight at 4 °C with a primary antibody against G6PI (1:200 in blocking) followed by incubation for 2h with the secondary antibody Alexa Fluor 555 goat anti-mouse (1:200; Molecular Probes, Eugene, OR, USA). Sections were washed in PBS and then incubated for 2h with primary antibodies against AMFR (1:100 in blocking) followed by incubation for 1 h at 37°C with the secondary antibody goat anti-rabbit IgG FITC (Novus Biologicals). All sections were counterstained with TO-PRO-3 (Molecular Probes). Negative controls were performed by omitting the primary antibodies. Specific fluorescence was acquired by a Leica TCS SP2 (Leica, Wetzlar, Germany) confocal laser-scanning microscope using an ×63 objective lens.

### Wound healing assay

2×10^5^ normal and tumor renal cells were seeded to create a confluent monolayer onto a six-well plate. Cells were incubated overnight at 37°C, 5% CO2, and then exposed to 2ng/μl of antibody anti-AMFR (Novus Biologicals) for 30 minutes or incubated in medium alone. A wound was manually created by scraping the cell monolayer with P200 pipette tip. Cells were washed four times with PBS and incubated with 2 mL of Keratinocyte Serum-Free Medium (KSFM), supplemented with 5ng/ml recombinant epidermal growth factor (rEGF), 50μg/ml bovine pituitary extract (BPE) (Gibco) and 30ng/ml cholera toxin (Sigma-Aldrich). A reference mark was created on the dish and a time 0 image was acquired. After 12 and 24 hours, additional images were taken in the matched region, and the wound-healing area was quantified with ImageJ software (http://rsbweb.nih.gov/ij/).

### *In vivo* chorioallantoic membrane (CAM) angiogenic and invasion assays

Fertilized White Leghorn chicken eggs were incubated at 37°C at constant humidity. On day 3 of incubation a square window was opened in the egg shell after removal of 2–3 ml of albumen so asto detach the developing CAM from the shell. The window was sealed with a glass and the eggs were returned to the incubator. Gelatin sponges (Gelfoam, Upjohn Company, Kalamazoo, U.S.A.) were cut to a size of 1 mm3 and placed on top of the growing CAM at day 8 incubation under sterile conditions according to Ribatti et al [36]. The sponges were then adsorbed with 2 μl of cell suspension of normal or tumor cells with medium alone or supplemented with antibody anti-AMFR (2 ng/μl). The angiogenic response was evaluated on day 12 of incubation after the implants by means of a stereomicroscope connected to an image analyzer system (Olympus Italia, Italy). Blood vessels entering the sponges within the focal plane of the CAM were counted by two observers in double blind fashion at a magnification of 50x.

For invasion assay CAMs were fixed in ovo in Bouin's fluid and processed for paraffin embedding. Then, 7-mm serial sections (10 sections per CAM) were cut parallel to the surface of the CAM, stained with hematoxylin-eosin, and the counts of infiltrating tumor cells infiltrating the CAM's mesenchyme cells were performed on 3 to 5 non-overlapping contiguous fields observed at 200 × magnification [37]. Means ± standard deviation (SD) were evaluated for all the parameters and the statistical significance of the differences between counts was determined by Student's t-test for unpaired data.

### Cell viability assay

Cell viability after exposure to 6-Aminonicotinamide (6-AN) (Sigma-Aldrich S.r.l., Milan, Italy) or to 6-AN and cis-Diamminedichloroplatinum(II) (cisplatin) 10 μM was assayed using the trypan blue dye exclusion and 3-(4,5-dimethylthiazol-2-yl)-2,5-diphenyltetrazolium bromide (MTT) assay. For the dye exclusion test and MTT assay, normal and tumor cells were seeded at a density of 1.5×10^5^ and 1.5×10^4^ cells in six-well and 96-well plates, respectively (Sigma-Aldrich S.r.l., Milan, Italy) and incubated overnight at 37°C, 5% CO2. In the first part of experiment, the cells were exposed to 250μM of 6-AN for 6, 10 and 24h, or incubated in medium alone. After several washes to remove 6-AN, the cells were incubated for 72h. In the second part of experiment, the cells were exposed to 250μM of 6-AN for 24h and then were treated with cisplatin 10 μM for 1h and 2h. After several washes to remove cisplatin the cells were incubated for 72h.

### siRNA transfection

Isolated normal and tumor renal cells were cultured at 2×10^5^ cells per well in a 12-well plate with Keratinocyte Serum-Free Medium (KSFM), supplemented with 5ng/ml recombinant epidermal growth factor (rEGF), 50μg/ml bovine pituitary extract (BPE) (Gibco) and 30ng/ml cholera toxin (Sigma-Aldrich). The transfection of siRNA was carried out using Lipofectamine 3000 (Life Technologies) in accordance with manufacturer's procedure. For each transfection, 50nM of siRNA G6PDH (Qiagen) were used. In transfection experiments, a mock-transfection control was performed by putting cells through the transfection procedure without adding siRNA. The validated nonsilencing siRNA sequence AllStars Negative Control siRNA (50 nM, Qiagen) was used as negative control. Each transfection experiment was performed in triplicate. After transfection, cells were incubated for 24h and 72h at 37°C in 5% CO2 and used, respectively, for total RNA extraction and cell viability assay.

### Real time PCR

Total RNA of transfected normal and tumor kidney cells was reverse transcribed with the High-Capacity cDNA Reverse Transcription Kit (Applied Biosystems Foster City, CA, USA), following the manufacturer's instructions. Quantitative real-time polymerase chain reactions (PCR) were performed using iQTM SYBR Green Supermix buffer (6mMMgCl2, dNTPs, iTaq DNA polymerase, SYBR Green I, fluorescein and stabilizers) (BIO-RAD Laboratories, Hercules, CA, USA). The primers used for G6PDH were 5′-GAGGCTGCAGTTCCATGATG-3′ and 5-'GACTCCTCGGGGTTGAAGAA-3′. Quantification of the mRNA levels was performed on a MiniOpticon Real-Time PCR detection system (BIO-RAD Laboratories). In the PCR reactions the following protocol was used: activation of the polymerase 95°C for 3 min, followed by 45 cycles of 95°C for 10 s, 60°C for 30 s. Melting curves were generated through 60 additional cycles (65°C for 5 s with an increment of 0.5°C/cycle). Gene expression results were obtained as average Ct (threshold cycle) values of triplicate samples. Expression was determined using the 2^−ΔΔCt^ method. Expression values were normalized to Glyceraldehyde 3-phosphate dehydrogenase (GAPDH).

### NADPH measurement assay

Primary tumor and normal cells were plated in duplicate six-well plates. Following treatment with 6-AN, cells were lysed in NADP/NADPH extraction buffer (Elite fluorimetric NADP/NADPH assay kit, e-ENZYME, Gaithersburg, MD, USA). In a 96-well plate, 50 μl extracted lysates were added to 50 μl NADPH recycling buffer. The plate was incubated at room temperature for 2h and measured at 560/610 Ex/Em. Fluorescence was normalized to the total protein levels of each sample.

### ROS measurement assay

Normal and tumor cells were seeded at a density of 2×10^5^ on glass coverslips and left to adhere overnight at 37°C in 5% CO2. In the first part of experiment, the cells were exposed to 250μM of 6-AN for 24h, or incubated in medium alone, as previously described. In the second part of experiment, the cells were exposed to 250μM of 6-AN for 24h and then were treated with 100 μM of ascorbic acid 2-phosphate (AA2P) for 24h. After several washes to remove AA2P the cells were incubated for 72h. To study mitochondrial superoxide generation, cells were stained with 5 μM MitoSOX red for 10 min at 37°C and washed three times before imaging. Cells were fixed using ice-cold 4% paraformaldehyde for 10 min at room temperature. Then, nuclei were revealed by counterstaining with TO-PRO-3 (Molecular Probes). Images were taken with a Leica TCS SP2 (Leica, Wetzlar, Germany) confocal laser-scanning microscope using an ×63 objective lens.

### Statistical analysis

Statistical calculations were performed with MedCalc 9.2.0.1 (MedCalc software, Mariakerke, Belgium) and PASW 18 software (PASW 18, SPSS, Chicago, Ill, USA). Global biochemical profiles were determined in human kidney tissue/tumor samples and compared across groups stratified by renal tissue pathology status. An estimate of the false discovery rate (q-value) was calculated to take into account the multiple comparisons that normally occur in metabolomic-based studies. Comparisons of metabolites median values between different groups were evaluated by Mann–Whitney U test. Receiver Operating Characteristic (ROC) curve analysis was performed to identify the G6PI, TKT and G6PDH protein expression cut-offs for survival stratification.

In the cancer-specific survival (CSS) analysis, patients still alive or lost to follow-up were censored, as well as patients who died from RCC-unrelated causes. Progression-free survival (PFS) was calculated from the date of surgery to the date of disease recurrence. Estimates of CSS and PFS were calculated according to the Kaplan-Meier method and compared with the log-rank test. Spearman's correlation was applied to evaluate associations between these markers and tumor stage/grade. A *P*-value of < 0.05 was considered statistically significant.

## SUPPLEMENTARY MATERIALS, FIGURES


