# Supplementary material for: Metabolomic profile of glycolysis and the pentose phosphate pathway identifies the central role of glucose-6-phosphate dehydrogenase in clear cell-renal cell carcinoma
Source: Oncotarget. 2015 Apr 14;6(15):13371–86. doi: 10.18632/oncotarget.3823 (PMC4537021; doi:10.18632/oncotarget.3823)
Supplement: Supplementary file 1 [file oncotarget-06-13371-s001.pdf]

Metabolomic profile of glycolysis and the pentose phosphate pathway identifies the central role of glucose-6-phosphate dehydrogenase in clear cell-renal cell carcinoma

Supplementary Material

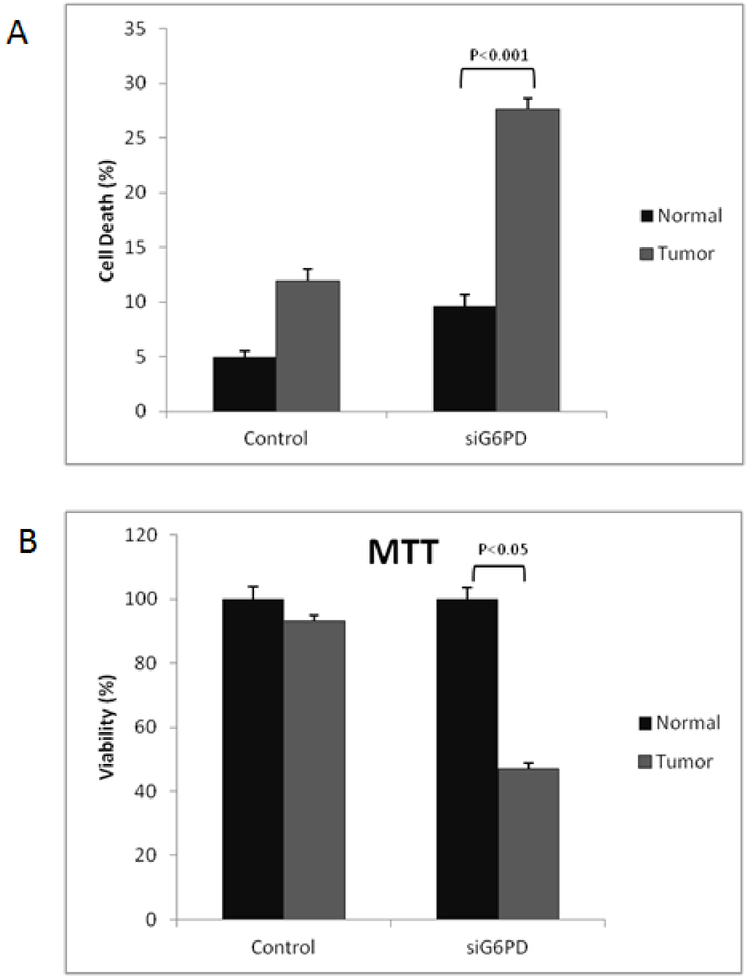

Supplementary Figure 1: Inhibition of G6PD suppresses renal cancer cell proliferation. (A, B) normal and tumoral cells were transfected whit siRNA targeting G6PD. After 72h transfection, cells were assayed (A) using trypan blue dye (Normal vs Tumor $p<0.001$) in and (B) MTT analysis (Normal vs Tumor $p<0.05$).

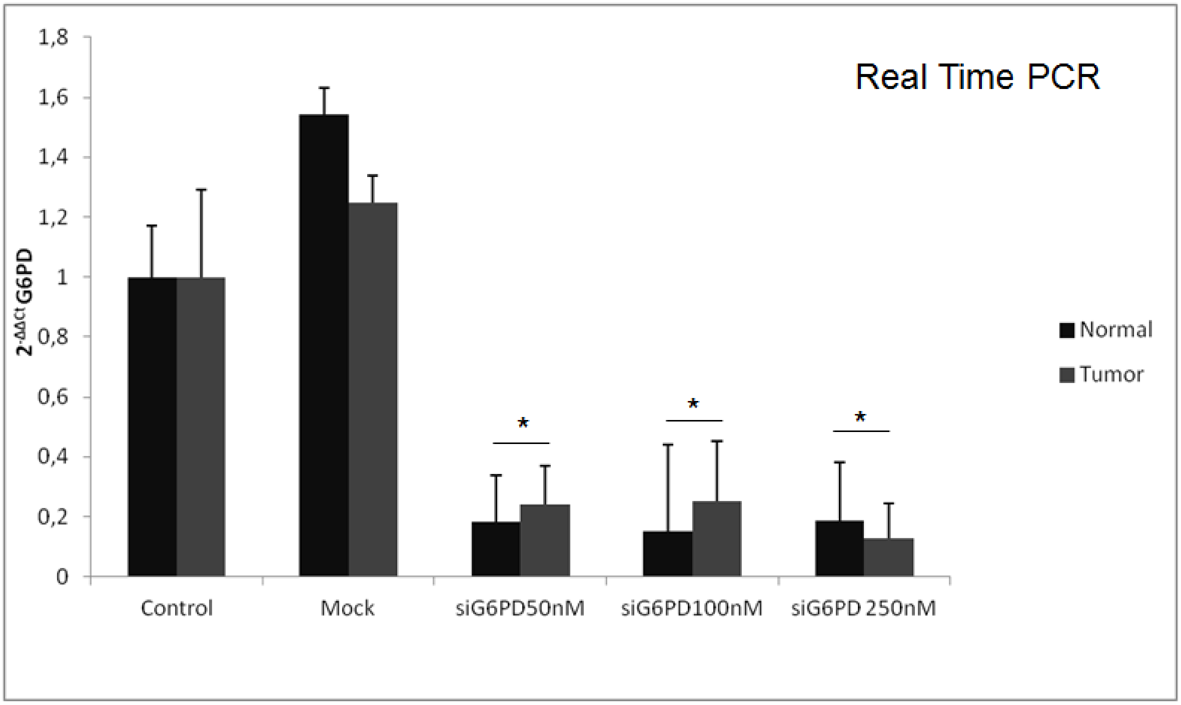

Supplementary Figure 2: Validation of the G6PDH silencing: Expression levels of G6PD were quantified using pRT-PCR. RNA expression was normalized to the expression of GAPDH. Levels of G6PDH were found to be significantly lower in normal and tumor cells treated with siG6PD (50, 100 and 250 nM siG6PDH), respect to control and mock conditions. The histogram represents the mean ± SEM. *P<0.01.
